# A Medical Causerie

**Published:** 1915-07-24

**Authors:** 


### A MEDICAL CAUSERIE.

The decision of the War Office to modify the rules governing the rejection of recruits on physical grounds—that is, so far as home service is concerned—will be generally approved by the medical profession. That a high standard for foreign service is necessary no sensible person can doubt, especially in view of the hardships to which the troops were exposed in the winter campaign. In the early and enthusiastic days there were many critics who claimed that men were being unnecessarily rejected, but little has been heard on this score recently. We know more of the realities of the situation than we did last autumn. But a defect which may render a recruit unsuitable for the conditions under which fighting is conducted in Flanders need not make him valueless for home service. Here, even if he does nothing else, he may liberate a more robust comrade, and may thus increase the fighting forces. The authorities have, perhaps somewhat tardily, recognised this position, and The Hospital may well acquiesce, seeing that it is the policy advocated in its columns so far back as September 12 last (p. 649).

Discussion is once more active on the question of the medical *Who's Who*, and the seemliness, or otherwise, of practitioners entering their names and decorations in this volume. When first arranged the scheme, it was understood, would only include certain selected persons, but this proposal raised a storm of protest. Now the circular asking for the desired particulars is issued to every practitioner, and the question of inclusion or exclusion rests with the individual himself. Apparently a considerable number of practitioners view the enterprise with disfavour, for there are many gaps in the volume, and it is not easy to see what purpose such a publication serves other than the desire for notoriety. But the odd thing is that some who condemn the medical volume yet contribute their own names to the ordinary non-medical *Who's Who*. Surely if professional modesty is hurt by the one, it is still more outrageously shocked by the other; and if personal advertisement is seemly, it would be more dignified openly to proclaim it rather than to secure it by a side-wind. In any event, if horses may be stolen by baronets and professors, more humble individuals will insist on their right to look over hedges. The whole question has a trumpery and petty aspect, though in essence it involves an important principle of professional conduct. If personal proclamations are permitted in one popular medium, with what countenance can they be censored in others?

The Universities, very appropriately, are doing something to sustain the view that there is work, other than that immediately directed to the war, which is yet worthy of recognition. The University of Glasgow has conferred the honorary degree of LL.D. on Dr. George S. Middleton, who, after many years of service, recently retired from the medical staff of the Glasgow Royal Infirmary. Dr. Middleton is one of the best-known consulting physicians in Scotland, and has made many contributions of considerable value in the several departments of clinical medicine. He is at present a lieutenant-colonel on the staff of one of the local military hospitals. A pleasing feature of the compliment paid to him by the University was the presentation of his new academic robes by his colleagues on the staff of the Royal Infirmary. Another honorary graduate is Major Robert McCarrison, of the Indian Medical Service, who has been made a D.Sc. by his Alma Mater, the Queen's University of Belfast. Sent on service some years ago to the extreme north of India, Major McCarrison conducted a most elaborate series of researches on the etiology of goitre, and recently he presented the results of these in a series of lectures to the Royal College of Physicians of London. At present he is on active service in Egypt, and his latest distinction will give pleasure to his numerous friends.

Another recognition, not this time from a University, which will be cordially endorsed is the presentation of the gold medal of the British Medical Association to Captain Arthur Martin-Leake, R.A.M.C., who, as everyone knows, was awarded the V.C. in the South African War, and now wears this distinction with an added bar for most conspicuous bravery and devotion to duty in the present campaign.

The pressure of the day and hour appears to be particularly severe at Bristol, if a judgment may be formed from some recent appointments. The western city has had many troubles since it attained to University rank, and just now it evidently has difficulties in filling the ranks of its junior teachers, for one and the same lady has recently been appointed to hold two such important offices as demonstrator in obstetrics, and teacher in ophthalmology, laryngology, and otology. The Royal Infirmary, too, has not escaped, for there another lady associates the functions of obstetric officer with the duties of ophthalmic house surgeon. Who, after this, can doubt that the women doctors are able to take advantage of the opportunities created by the war?

At an exhibition now open in London a special lecture on "Children and the Fly Peril," illustrated by lantern-slides and other realistic demonstrations, is well calculated to reinforce the campaign which the bad boy of the family naturally conducts against all winged insects and creeping things. Models which display the capacity of the fly-mother to multiply her pernicious brood, and the inclination of the whole family to contrive various arrangements prejudicial to the human intestine, will hardly fail to impress the youthful mind with the conviction that the fly is *hostis humani generis*, and thus new vigour will be lent to the art of destruction. What by the parental mind was formerly considered an evidence of original sin is now found to be a praiseworthy protective mechanism against some of the ills which beset society. Thus Wisdom is justified of her children.

